# High‐Value Transformation of Rice‐Derived Bioactive Peptides for Multiscenario Applications: From AI‐Aided Design to Molecular Modification

**DOI:** 10.1155/ijfo/5561598

**Published:** 2026-06-09

**Authors:** Tianle Yao, Jie Yao, Qian Zhang, Mengtian Huang, Yu Han, Qin Li, Mengyuan Zhang, Li Li

**Affiliations:** ^1^ Wuhan City Polytechnic, Wuhan, China; ^2^ Hubei Key Laboratory of Resource Utilization and Quality Control of Characteristic Crops, College of Life Science and Technology, Hubei Engineering University, Xiaogan, China, hbeu.cn; ^3^ College of Biological and Food Engineering, Hubei Minzu University, Enshi, China, hbmy.edu.cn

**Keywords:** bioactive peptide, functional applications, functional enhancement, high-value development, rice protein

## Abstract

Rice‐derived bioactive peptides are gaining attention for their nutritional value, low allergenicity, and multifunctional health benefits. However, large‐scale utilization remains constrained by challenges such as low protein extraction efficiency, limited peptide yields, and poor structural stability. This review summarizes recent advances in the valorization of rice proteins into functional peptides, with a focus on emerging technologies including enzymatic hydrolysis optimization, AI‐assisted peptide design, and polyphenol‐mediated molecular modification. These approaches offer solutions to improve thermal stability, reduce aggregation, and enhance bioactivity. The review further explores its multiscenario applications in AI‐driven design and modification, cosmetics, biomedicine, and functional foods, where rice peptides demonstrate promising application potential in antioxidant defense, blood pressure regulation, collagen synthesis, and heavy metal adsorption. By integrating molecular design, processing innovation, and application development, this work provides a comprehensive perspective on the high‐value transformation of rice by‐products and supports the advancement of sustainable rice peptide technologies.

## 1. Introduction

Rice (*Oryza sativa* L.) serves as a staple food for approximately 3.5 billion people globally, with consumption patterns exhibiting strong regional concentration [1]. According to the International Rice Research Institute (IRRI) (2024), annual global rice consumption exceeds 521 million tons, with China (148 million tons), India (112 million tons), and Southeast Asian countries (a combined 105 million tons) accounting for more than 70% of the total [2]. The large‐scale processing of rice generates substantial quantities of by‐products. The Food and Agriculture Organization (FAO) estimates that over 140 million tons of rice bran and broken rice are produced annually, containing around 8.6 million tons of high‐purity protein [3]. Nevertheless, the global utilization rate of rice protein remains below 30%, with approximately 68% of these by‐products relegated to low‐value applications such as animal feed or industrial adhesives, resulting in an estimated annual economic loss exceeding $12 billion [4].

**Figure 1:**
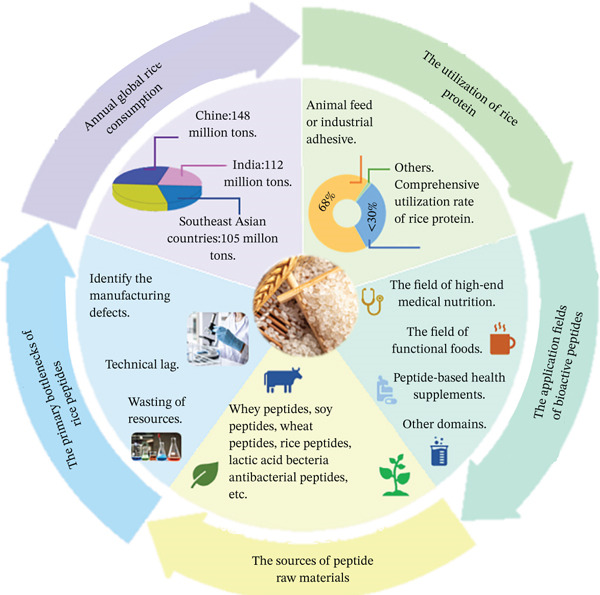
Global development map of rice protein resources and active peptide industry.

Concurrently, the bioactive peptide market is undergoing rapid and diversified expansion (Figure 1). According to Grand View Research (2024), the global market is expected to grow from $11.25 billion in 2023 to $24.2 billion by 2030, at a compound annual growth rate (CAGR) of 11.6% [5]. Market demand is regionally stratified: North America dominates the medical nutrition sector, where peptide applications are subject to strict regulatory oversight such as FDA GRAS certification (e.g., 21 CFR 184.1983 for whey peptides) and the EU Novel Food Regulation (EC 2015/2283) [6]. Japan leads the functional food sector under the FOSHU certification system, where antihypertensive and antioxidant peptides account for more than 65% of the market (JHNFA 001‐2022) [7]. In contrast, emerging markets such as Brazil and India are experiencing accelerated growth driven by relaxed regulatory policies, including ANVISA RDC 27/2010 and India′s 2023 FSSAI regulations specifying intake limits for soy and rice peptides [8].

The diversification of peptide sources has prompted the implementation of stringent national quality and safety standards. Animal‐derived peptides—such as whey peptides, which account for 45% of global supply—must meet ISO 20776:2020 for antimicrobial efficacy and HACCP certification for collagen peptides in Japan [9]. Plant‐derived peptides are led by soy peptides (minimum 80% peptide content per GB 31645‐2018 in China), whereas wheat peptides are regulated under the EU′s nutrition claims legislation (EC 1924/2006) [10]. Microbial fermentation is emerging as a novel production route, with yeast extract peptides regulated under FDA 21 CFR 172.325 and bacteriocin‐like peptides from lactic acid bacteria governed by IDF 189:2020 [11].

Against this backdrop, rice‐derived bioactive peptides have emerged as a strategically valuable resource owing to their unique advantages. Their low allergenicity is supported by both US FDA allergen threshold data (< 1.0‐*μ*g/g IgE binding; GRN 000986) and EFSA sensitization guidance (2021/C 228/01) [12]. Additionally, their essential amino acid index (EAAI = 0.82) exceeds the FAO/WHO reference standard (0.80) [13]. Despite these favorable attributes, industrial utilization remains limited. According to Fan et al., rice peptides represent only 1.8% of the global bioactive peptide market, trailing far behind whey (45%) and soy peptides (22%) [14]. The primary bottleneck lies in processing inefficiencies: Conventional alkaline extraction methods (NaOH 0.1–0.15 M) result in high protein denaturation rates (> 60%) and low tryptophan retention (< 40%), whereas enzymatic hydrolysis yields remain below 18%—approximately 40 percentage points lower than those achieved with whey proteins [15]. Bridging the gap between resource potential and technological limitations is therefore imperative for the sustainable advancement of rice protein valorization within the global food system.

## 2. Classification of Rice Proteins and Derived Bioactive Peptides

A systematic understanding of rice varietal traits and protein composition forms the basis for the high‐value development of rice‐derived bioactive peptides [16]. Rice is typically classified into four types—japonica, indica, glutinous, and pigmented—based on grain morphology and starch profile, each differing in protein content and extraction characteristics [17]. Corresponding proteins are grouped by solubility into albumin, globulin, prolamin, and glutelin, whose structural features affect enzymatic hydrolysis efficiency [18]. The resulting peptides are further categorized by bioactivity and molecular properties—such as antioxidant, angiotensin‐converting enzyme (ACE) inhibitory, or antimicrobial functions—establishing a coherent framework linking rice type, protein structure, and peptide function for targeted applications.

### 2.1. Rice Classification

Rice, widely cultivated across the globe, can be broadly categorized into four types based on grain morphology, starch composition, and botanical characteristics: japonica, indica, glutinous, and pigmented rice [16, 17]. These classifications reflect not only morphological traits but also key differences in chemical composition and processing behavior (Table 1) [19].

**Table 1:** General classification table of rice types and protein characteristics.

Classification dimension	Japonica rice	Indica rice	Glutinous rice	Pigmented rice
Botanical classification	*Oryza sativa* subsp. *japonica*	*Oryza sativa* subsp. *indica*	*O*. *sativa* subsp. *japonica* var. glutinous or *O*. *sativa* subsp. *indica* var. glutinous	*O. sativa* subsp. *japonica* or *indica*.
Grain morphology	Short and round, with a length‐to‐width ratio of less than 2.0, transparent and hard.	Long and slender, with a width‐to‐length ratio greater than 3.0, and a soft texture.	Waxy white in color, opaque, without linear starch endosperm.	Black/red/purple seed coat, with thickened chaff layer.
Starch composition	Straight‐chain starch 15%–20%	Straight − chain starch > 20*%*	Branching starch≈100%	The seed coat contains pigments such as anthocyanins and proanthocyanidins.
Steaming and boiling characteristics	High viscosity and prone to agglomeration.	Low viscosity, grain dispersion.	High viscosity elasticity, high glossiness.	Compact and with a distinct fibrous texture.
Representative varieties	Koshihikari rice, Northeast rice.	Jasmine rice, Basmati rice.	Wuchang glutinous rice.	Black fragrant japonica rice, Donglan black rice.
Total protein contents	7%–9% (dry basis)	6%–8% (dry basis)	Similar to the corresponding japonica and indica varieties.	8%–10% (dry basis, increased bran layer)
Various protein contents	Albumin (2%–8%, water‐soluble) globulin (5%–10%, dilute salt solution) proteinase K (5%–10%, 70% ethanol) glutelin (60%–80%, dilute acid/base reducing agent)	The proportion of albumin is higher compared with regular rice.	For the corresponding variety.	Compared with regular rice, the presence of phenolic compounds increases the difficulty of extraction. Therefore, a peptide‐phenolic compound synergistic system can be developed.
Protein component characteristics	Gluten accounts for 60%–80% of the total.	The proportion of albumin is slightly higher (5%–10%).	The protein content is similar, and the amylose affects the rheology.	Gluten is the main component, and phenolic compounds form complexes with proteins.
Other properties	—	—	Branching starch is nearly 100%.	Rich in anthocyanins/tannins/phenols, dietary fiber, and minerals.

Japonica rice, exemplified by varieties such as Koshihikari from Japan and cultivars from Northeast China, features short, round grains with high translucency and firm texture [20]. Its moderately low amylose content (15%–20%) contributes to a cohesive, sticky consistency upon cooking [21]. In contrast, indica rice, including Thai Jasmine and Indian Basmati, is defined by long, slender grains (length − to − width ratio > 3.0) and higher amylose content (typically > 20%), resulting in a looser, less sticky texture when cooked [22].

Glutinous rice, a distinct subspecies, contains nearly 100% amylopectin with negligible amylose [23]. Its grains appear waxy and opaque, producing high gloss and elasticity after cooking—traits desirable in certain traditional foods [24]. Pigmented rice varieties, such as black, red, and purple rice, are characterized by the accumulation of natural pigments in the bran [25]. Black and purple rice are rich in anthocyanins (e.g., cyanidin‐3‐glucoside), whereas red rice contains abundant proanthocyanidins and tannins [26].

Beyond their visual appeal, pigmented rice types possess a thickened bran layer enriched in dietary fiber, minerals, and phenolic compounds such as ferulic acid and p‐coumaric acid [27]. These polyphenols can interact with rice proteins through covalent and noncovalent mechanisms to form stable protein–polyphenol complexes [28]. Such interactions significantly impact the extraction efficiency and structural properties of rice proteins during downstream processing, offering both opportunities and challenges in the development of value‐added products.

### 2.2. Protein Classification in Rice

Rice proteins, as the fundamental precursors of bioactive peptides, show considerable diversity in both content and composition across different rice cultivars. In polished rice, total protein content typically ranges from 6% to 9% on a dry basis and can be classified into four solubility‐based fractions: albumin, globulin, prolamin, and glutelin [29].

Albumins, soluble in water, account for approximately 2%–8% of total rice protein [30]. These low‐molecular‐weight proteins are enriched in sulfur‐containing amino acids such as methionine and cysteine [31]. Globulins, which dissolve in dilute salt solutions, contribute about 5%–10% of total protein and are characterized by a broad molecular weight distribution and a relatively high abundance of basic amino acids like arginine [32].

Prolamins, soluble only in 70%–80% ethanol, make up 5%–10% of total protein and are notably rich in hydrophobic amino acids (up to 42%) [33]. Their secondary structures contain a high proportion of *β*‐sheets, often exceeding 55%, whereas dense intramolecular disulfide linkages lead to extremely poor water solubility—typically under 5% at room temperature [34]. This limited solubility presents a significant barrier to enzymatic hydrolysis and bioactive peptide generation.

Glutelins represent the predominant storage proteins in rice, comprising 60%–80% of total protein [35]. They are extractable only under extreme pH conditions—either acidic (pH < 4) or alkaline (pH > 10)—and often require reducing agents such as mercaptoethanol to break disulfide bonds [36]. Structurally, glutelin is composed of acidic (30–39 kDa) and basic (19–25 kDa) subunits joined by disulfide linkages, forming high‐molecular‐weight polymers that serve as the primary substrate for peptide hydrolysis [37].

The distribution of these protein fractions varies notably across rice types. Japonica rice tends to have a higher total protein content (7%–9%), with glutelin as the dominant fraction [20]. Indica rice exhibits slightly lower protein levels (6%–8%) but an elevated proportion of prolamin [22]. Glutinous rice presents a comparable protein profile to its japonica or indica counterparts, but its nearly 100% amylopectin content significantly alters processing rheology [23]. Pigmented rice varieties, such as black and red rice, often possess elevated protein content (8%–10% or more), primarily due to the thickened bran layer [25]. Glutelin remains the dominant fraction, but the presence of abundant phenolics—such as anthocyanins in black rice or tannins in red rice—promotes the formation of stable protein–polyphenol complexes through covalent bonding or hydrogen interactions [36]. Although these complexes complicate protein extraction, they also offer a unique foundation for developing synergistic systems that combine peptide bioactivity with phenolic antioxidant functionality.

### 2.3. Classification of Bioactive Peptides from Rice Protein

Rice‐derived bioactive peptides are short‐chain oligopeptides, typically comprising 2–20 amino acid residues, generated through enzymatic hydrolysis or microbial fermentation [38]. These peptides often feature hydrophobic residues—such as phenylalanine, tryptophan, and leucine—at their C‐terminus, with occasional N‐terminal acetylation, structural motifs closely linked to their bioactivities (Table 2). Depending on their functional targets and precursor protein origins, rice peptides exhibit a wide spectrum of biological effects, including antioxidant, ACE inhibitory, antimicrobial, gut‐regulatory, and collagen metabolism–modulating activities [39].

**Table 2:** Classification table of bioactive peptides in rice protein.

Classification dimension	Specific category	Sources	Structural characteristics	Function
According to the core biological functions and the sources of precursor proteins	Antioxidant peptides	The enzymatic breakdown products of gluten.	Representative peptide segment GYFNNL, with an asparagine–leucine sequence at the carboxyl terminus.	By directly eliminating reactive oxygen free radicals through the carbon‐terminal sequence, the Keap1–Nrf2–ARE signaling pathway is activated, and the expression of antioxidant enzymes is upregulated.
	ACE inhibitory peptides	Hydrolyzates of gluten and prolamin.	Typical sequence: RRWQWRF, with phenylalanine at the carboxyl terminus.	By relying on the strong van der Waals interaction between the carboxyl‐terminal phenylalanine and the hydrophobic pocket S2 of the ACE active center, it competitively blocks the conversion of Angiotensin I to Angiotensin II.
	Antimicrobial peptides	Hydrolysis of prolamin and gluten results in.	Having amphiphilic structure	By utilizing electrostatic interaction to target the bacterial cell membrane, the membrane integrity is disrupted, leading to cell death.
	Intestinal regulatory peptides	The hydrolyzed components of gluten and prolamin.	Higher sequence conservation.	Perception of the metabolic products of intestinal microorganisms regulates the secretion of hormones such as GLP‐1 and PYY.
	Collagen metabolism‐related peptides	Enzymatic breakdown products of gluten and prolamin.	Such as prolyl‐hydroxyproline dipeptide, glycyl‐prolyl‐hydroxyproline tripeptide.	By specifically binding to the transforming growth factor‐*β* receptor, it activates the phosphorylation of the Smad3 signaling protein.

According to molecular weight and structural characteristics	Small molecule peptides (with a molecular weight of less than 1 kDa)	The deep enzymatic hydrolysis products of albumin, globulin, solubilin and glutenin.	Low molecular weight, weak steric hindrance.	It has a high affinity for the PepT1 transporter in intestinal epithelial cells and a high oral bioavailability.
	Cyclic peptides	It is obtained through artificial design of cyclization based on the hydrolyzed fragments of glutenin or gliadin.	Rigid conformation.	It can resist protease degradation and enhance the specificity of target binding.

According to the specificity of the action site	Intestinal‐targeting peptides	Albumin, globulin and some gluten proteins.	There are RGD sequences, CendR sequences, and so on.	Through acid‐resistant design or mucosal adhesion properties, it ensures that the active ingredients are locally concentrated in the intestinal tract and act on specific receptors.
	Peptides for penetrating the blood–brain barrier	Gluten and prolamin.	Such as the angiopep‐2 sequence	Targeting brain capillary endothelial cells with LRP1 mediates nonglycoprotein‐dependent endocytosis.

Antioxidant peptides, primarily derived from glutelin hydrolysates, exemplify this functional diversity [40]. For instance, the representative peptide GYFNNL (molecular weight 789 Da) can directly scavenge reactive oxygen species (ROS) via its C‐terminal Asn–Leu motif (binding constant: 1.2 × 10^5^ M^−1^), while also activating the Keap1–Nrf2–ARE pathway, leading to a threefold increase in HO‐1 expression in HepG2 cells. Its DPPH radical‐scavenging EC_50_ is 0.8 mg/mL, and its antioxidant efficiency in functional beverages reaches 90.15*%* ± 1.02*%* [41]. ACE inhibitory peptides, mainly originating from glutelin and prolamin hydrolysates, often contain terminal phenylalanine residues that interact with the S2 hydrophobic pocket of ACE via strong van der Waals forces (accounting for 65% of binding energy) [42]. A typical example is the RRWQWRF peptide (1103 Da), with an IC_50_ as low as 0.08 *μ*M (compared with 4.8 *μ*M for l‐*β*‐Asp‐Pro), demonstrating dose‐dependent antihypertensive effects in vivo [43].

Antimicrobial peptides, typically amphiphilic in structure, are produced from prolamin and glutelin hydrolysates [44]. These molecules interact electrostatically with bacterial membranes, disrupting membrane integrity and inducing lysis. Optimized variants have significantly reduced the minimum inhibitory concentration (MIC) against methicillin‐resistant *Staphylococcus aureus* (MRSA) from 64 to 16 *μ*g/mL [45]. Peptides with gut‐regulatory functions are widely found in hydrolysates of both glutelin and prolamin [46]. These peptides interact with microbial metabolites such as short‐chain fatty acids and modulate hormone secretion, including GLP‐1 and peptide YY (PYY), thereby contributing to appetite regulation, energy homeostasis, and intestinal barrier integrity [47]. Although not predominant in rice, collagen metabolism–associated peptides such as Pro–Hyp and Gly–Pro–Hyp analogs have been identified in glutelin and prolamin digests [48]. These peptides exhibit specific bindin06g to TGF‐*β* receptors (binding energy: −8.2 kcal/mol), activate Smad3 phosphorylation (increasing the p‐Smad3/Smad3 ratio by 1.8‐fold), and directly stimulate transcriptional activity of the COL1A1 gene (2.3‐fold enhancement), thereby promoting fibroblast‐mediated collagen synthesis [49].

In terms of structural properties, rice‐derived peptides can be further divided into linear small peptides and cyclic peptides. Small peptides, typically < 1 kDa, result from extensive enzymatic hydrolysis of albumin, globulin, prolamin, and glutelin. Owing to their low molecular weight and minimal steric hindrance, they display high affinity for the PepT1 transporter in intestinal epithelial cells (Km = 0.15 mM), exhibit enhanced oral bioavailability, and possess a plasma half‐life of up to 6 h, making them ideal carriers for precision nutrition delivery [50]. In contrast, cyclic peptides are typically designed by cyclizing fragments from glutelin or prolamin [51]. Their rigid conformations confer protease resistance and improved target‐binding specificity. For example, cyclic probes targeting the gastric cancer stem cell marker LGR5 show threefold higher fluorescence intensity in cancer cells than in normal cells, indicating their potential utility in high‐resolution diagnostics and therapeutic delivery systems [52].

Target‐specific functionality further refines their application, including intestinal‐targeting and blood–brain barrier (BBB)–penetrating peptides [53]. Intestinal‐targeting peptides, derived from albumin, globulin, and to some extent glutelin, are designed for acid resistance or mucosal adhesion to ensure localized delivery and interaction with specific receptors such as FFAR2/3 [54]. BBB‐penetrating peptides such as angiopep‐2, derived from glutelin and prolamin, can mediate clathrin‐independent transcytosis by targeting low‐density lipoprotein receptor–related Protein 1 (LRP1) on brain endothelial cells [55]. This mechanism enhances the BBB permeability of peptide‐loaded nanoparticles from less than 5%–28%, significantly prolonging median survival in glioma‐bearing animal models by 67% [56].

## 3. Analysis of the Core Advantages of Rice Active Peptides as an Emerging Technology Carrier

**Figure 2:**
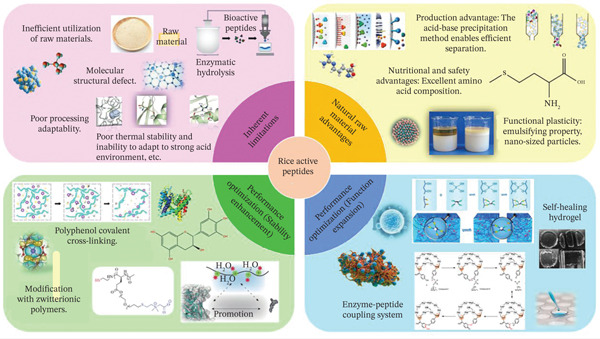
Core performance analysis chart of rice active peptides.

Rice‐derived bioactive peptides exhibit a unique combination of limitations and advantages that position them as promising carriers in emerging technological applications. Current processing approaches often suffer from low raw material utilization—typically below 40%—as well as structural instability resulting from peptide aggregation, which compromises functional efficacy. In contrast, rice proteins offer distinct benefits as plant‐based precursors, including minimal allergenicity, a balanced essential amino acid profile, and high compatibility with molecular engineering and interdisciplinary modification strategies [57]. Addressing these issues through a framework of bottleneck identification, intrinsic advantage optimization, and targeted technological innovation provides a pathway for unlocking the full functional and economic potential of rice peptides in high‐value applications such as functional foods, pharmaceuticals, and biomaterials (Figure 2).

### 3.1. Inherent Limitations of Rice Active Peptides

Despite increasing academic and industrial interest, the development and utilization of rice‐derived bioactive peptides remain in the early stages, with applications largely confined to low value‐added products [58]. The full industrial potential of these peptides has yet to be realized. This limitation stems from two core challenges: the inefficiency of rice protein extraction and processing technologies, and the intrinsic physicochemical characteristics of the peptides themselves [38].

From a raw material perspective, the low efficiency in utilizing rice proteins severely hampers the scalability and economic viability of peptide production. In the food sector, approximately 68% of rice bran protein is still used only as a feed additive [59], and the overall utilization rate of crude protein remains below 40% [60]. In the industrial sector, rice proteins are primarily exploited for their film‐forming properties in applications such as paper‐sizing agents [61], but the market for such products is expanding at a modest annual growth rate of only 2.3% [62]. The most critical technical bottlenecks appear at several stages: Conventional alkaline extraction methods result in protein denaturation rates exceeding 60%, severely compromising the integrity of functional domains and reducing subsequent enzymatic hydrolysis efficiency [63]. Even when enzymatic methods are employed—for instance, using neutral proteases under optimized conditions—the peptide yield from rice protein typically remains low, ranging from 13% to 16% [64], reflecting substantial resource wastage. These limitations contribute to a low overall value across the production chain, with limited profitability from rice bran protein processing, which fails to reflect its potential high economic value [65].

At the molecular level, rice‐derived bioactive peptides exhibit significant limitations in terms of structure and processing adaptability, which represent key barriers to their advancement into high value‐added applications [66]. Structurally, although these peptides exhibit functional diversity, they frequently retain hydrophobic regions that become problematic at higher concentrations (e.g., > 5 mg/mL), where exposed hydrophobic residues tend to self‐associate [67]. Dynamic light scattering (DLS) measurements show that particle size can increase dramatically from the nanoscale to nearly 100 nm, indicating significant aggregation. This behavior leads to reduced solubility and decreased bioavailability, which seriously compromises their performance in aqueous formulations such as functional beverages or injectable preparations.

Processing adaptability presents another major challenge. Rice proteins generally exhibit a lower denaturation temperature compared with soy protein isolates by approximately 12°C, suggesting inferior thermal stability [68]. More critically, rice peptides tend to aggregate and lose activity under extreme pH conditions, particularly in acidic environments (pH < 3). This instability is closely related to the surface charge characteristics of the source protein (with an isoelectric point around 4.5) and the ionization behavior of the peptide [69]. In highly acidic conditions, charge inversion may occur, leading to irreversible aggregation.

In summary, the industrial application of rice bioactive peptides is constrained by dual bottlenecks [58]. On one hand, the low extraction and utilization efficiency of rice proteins limits both the economic feasibility and scalability of peptide production [68]. On the other hand, the inherent challenges in solubility (especially under high‐concentration conditions) and environmental stability (e.g., thermal and pH tolerance) of the peptides themselves represent major obstacles to their deployment in high value‐added, technologically advanced applications.

### 3.2. Rice Active Peptides′ Inherent Advantages and Technical Empowerment Directions

The potential of rice‐derived bioactive peptides as next‐generation functional carriers lies in the synergistic integration of their intrinsic biological properties with modern technological innovations. Rice proteins, as the raw material, offer distinct advantages rooted in their natural origin: Their isoelectric point (pI ≈4.5) enables low‐energy, environmentally friendly extraction processes; they possess a well‐balanced essential amino acid profile, including sulfur‐containing amino acids at levels approximately 18% higher than those found in soy protein; and they exhibit extremely low allergenicity—less than one‐twentieth that of whey protein—making them particularly suitable for sensitive populations [70]. These natural benefits serve as a robust foundation for advanced molecular engineering. Recent progress in covalent modification techniques, such as polyphenol conjugation, has markedly enhanced thermal stability (with denaturation temperatures increased by up to 35%), whereas computer‐aided peptide design has significantly improved functional performance, exemplified by a 75% reduction in the MIC of designed antimicrobial peptides [71]. Collectively, these advancements reflect a strategic shift toward enhancing molecular stability, elevating bioactivity, and broadening functional applications. Together, they establish a coherent development trajectory characterized by “natural advantage as foundation, technological empowerment for added value.”

### 3.3. Advantages of Rice Active Peptides as Natural Raw Materials

The core competitiveness of rice‐derived bioactive peptides originates primarily from the exceptional quality of their plant‐based source—rice protein—which provides a solid foundation for both peptide production and functional performance [72]. Moreover, rice peptides themselves inherit and even enhance several favorable characteristics.

One of the major production advantages lies in the extractability and process adaptability of rice protein as a precursor. Its isoelectric point is significantly lower than that of most other cereal proteins, enabling efficient separation through simple acid–base precipitation methods. This characteristic also makes it highly compatible with membrane separation technologies [70]. For instance, when ultrafiltration membranes are applied to rice protein hydrolysates, the membrane flux is approximately 40% higher than that observed for corn protein hydrolysates, substantially reducing the energy consumption and equipment costs in large‐scale production of rice peptides.

In terms of nutrition and safety, rice peptides demonstrate outstanding inherent advantages that support their development into high value‐added products. Nutritional balance is a key attribute, stemming from the excellent amino acid profile of the source protein. The essential amino acid index (EAAI = 0.82) of rice protein closely approaches the FAO/WHO recommended standard (0.85), with notably higher levels of sulfur‐containing amino acids compared with soy protein [13]. Peptides generated via enzymatic hydrolysis are more readily absorbed by the human body and effectively deliver these amino acid benefits [73]. Particularly important is their extremely low allergenicity, which stands out as one of the most critical safety attributes of rice peptides. Quantitative analyses indicate that allergen levels in rice protein are only a small fraction of those found in whey protein. Furthermore, enzymatic hydrolysis typically disrupts potential allergenic epitopes, making rice peptides an ideal low‐allergen protein source for high‐sensitivity consumer groups, such as in infant formula and foods for special medical purposes [74].

Functional plasticity is another key characteristic that underpins the broad application prospects of rice peptides. These peptides retain some interfacial activity of their parent proteins and may expose additional functional groups upon hydrolysis, thereby enhancing properties such as emulsification. More importantly, their ability to self‐assemble at the nanoscale opens up considerable potential in functional material applications [75]. Under specific processing conditions, rice peptides or their intermediate precursors can form nano‐sized particles with high encapsulation efficiencies for bioactive compounds [76]. This remarkable self‐assembly behavior provides a robust molecular basis for constructing highly stable and high‐loading delivery systems for functional ingredients [77].

Taken together, rice‐derived peptides benefit not only from the accessibility, nutritional balance, and safety of high‐quality plant proteins, but also exhibit intrinsic advantages including low allergenicity, high bioavailability, and strong functional plasticity—especially their superior potential in self‐assembled delivery systems [75, 77]. These attributes collectively establish a strong natural foundation for their role as emerging carriers in advanced technological applications.

### 3.4. Optimization Path of Rice Active Peptide Performance

**Figure 3:**
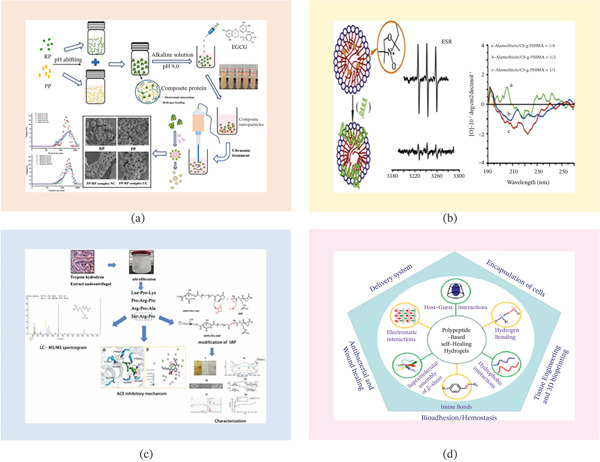
Optimization pathway for rice active peptide properties. (a) Structural and functional characteristics of ultrasound‐modified rice–peanut protein/EGCG ternary composite nanoparticles [78], (b) assembly characteristics of rice peptide‐based membrane peptides mediated by pSBMA‐grafted chitosan [79], (c) optimization of sustained‐release antihypertensive properties of DSPE‐PEG modified ACE inhibitory peptide [80], and (d) design and biomedical functional expansion of rice peptide‐based self‐healing hydrogels [81].

To overcome the inherent limitations of rice‐derived bioactive peptides in terms of stability, functional efficacy, and application breadth, and to fully unlock their potential as emerging carriers for advanced technologies, recent research has increasingly relied on interdisciplinary approaches to implement a series of deep functionalization strategies (Figure 3).

In terms of stability enhancement, polyphenol‐mediated covalent cross‐linking has proven highly effective in improving environmental tolerance [82]. For example, (‐)‐epigallocatechin gallate (EGCG) can form stable covalent adducts with amino groups on the peptide side chains, leading to the formation of extended conjugated structures. This modification significantly increases the thermal denaturation temperature from 68°C to 92°C and induces a red shift in ultraviolet (UV) absorption from 280 to 320 nm. HPLC‐MS analysis confirmed that EGCG primarily forms Schiff base adducts with the *ε*‐amino group of lysine in the peptide chain (m/z 785.2 → 469.1). Molecular dynamics simulation further revealed that the binding free energy (*Δ*G) of the crosslinked peptide‐EGCG complex decreased by −15.3 kcal/mol, and the conformational stability was significantly improved (RMSD reduced from 0.38 to 0.22 nm), endowing the material with enhanced antioxidant and photostability [78]. Preliminary economic evaluation showed that the EGCG addition (10% *w*/*w* based on protein mass) increased raw material costs by approximately 15%, but the overall industrialization advantage was achieved due to a 30% reduction in cyclodextrin usage and cold chain costs.

By covalently grafting hydrophilic poly(sulfobetaine methacrylate) (pSBMA) via amide bonds onto peptide termini, the resulting structure—featuring equimolar quaternary ammonium and sulfonate groups—forms a dense hydration shell and induces strong steric hindrance and electrostatic repulsion [83]. DLS analysis revealed that at 5 mg/mL in physiological buffer (pH 7.4), the average hydrated particle size decreased from 35.2 ± 3.5 nm to 7.8 ± 0.9 − nm postmodification, with a polydispersity index (PDI) maintained at 0.08 ± 0.02. Cryo‐EM (Thermo Fisher Glacios) imaging confirmed that the modified bioactive peptides presented as uniformly dispersed spherical nanoparticles (diameter 8.2 ± 1.3 nm), whereas unmodified peptides formed aggregates > 100 nm. Isothermal titration calorimetry (ITC) (MicroCal PEAQ‐ITC) quantification showed that pSBMA modification decreased the enthalpy change (*Δ*H) of intermolecular hydrophobic interactions in peptides from −12.3 ± 0.8 kcal/mol to −4.3 ± 0.5 kcal/mol [84]. The aggregation inhibition rate exceeded 85%, and circular dichroism (CD) spectra confirmed preservation of secondary structure integrity. This approach dramatically improves solubility, colloidal stability, and bioavailability at therapeutic concentrations [79].

From a functionality‐expansion perspective, intelligent response systems are becoming a new focal point. Self‐healing hydrogels, for instance, utilize dynamic covalent bonding to cross‐link peptide molecules, achieving over 95% mechanical recovery within 5 min of damage. These hydrogels also demonstrate excellent biocompatibility (cell viability > 90*%*) and injectability (shear − thinning index > 10), supporting their potential use in tissue engineering scaffolds [81]. Even more innovative is the development of enzyme–peptide conjugate systems, in which specific enzymes (e.g., glucose oxidase) are covalently linked or coimmobilized with functional peptides (e.g., antimicrobial or antioxidant peptides) onto protein‐based carriers to create autonomous functional units [85]. In such systems, glucose oxidase catalyzes the in situ generation of H_2_O_2_ from glucose, which not only exhibits direct antimicrobial effects but also activates the conjugated antioxidant peptides to scavenge excess ROS. Simulated gastrointestinal transit experiments (USP II dissolution apparatus, Sotax AT7) showed that the retention rate of bioactive peptides exceeded 90% in gastric environment (0.1‐M HCl, pH 2.0, 37°C, 2 h), whereas intestinal environment (PBS pH 6.8) triggered responsive release with 85% release rate within 2 h. Serum stability experiments (37°C, 10% FBS) indicated a half‐life *t*_1/2_ = 24 h (free peptides *t*_1/2_ = 6 h). This results in a seamlessly integrated “sense–respond–actuate” cascade with combined antibacterial and antioxidative functionalities, significantly expanding the application scope of rice peptides in smart packaging and biomedical platforms [86].

## 4. Extraction of Rice Protein and Preparation of Bioactive Peptides

This chapter systematically explains the full‐chain technology system for rice protein extraction and bioactive peptide preparation: from protein extraction methods such as alkali extraction and acid precipitation, enzymatic hydrolysis‐assisted and physical strengthening to solve the problem of starch coating, to the controlled hydrolysis process of active peptides with enzymatic hydrolysis as the core, and simultaneously analyzes the applicability of alternative pathways such as microbial fermentation and chemical hydrolysis, to construct a complete technology chain of “raw material pretreatment–protein extraction–peptide preparation–refining optimization,” providing a process paradigm for the high‐value transformation of rice protein resources (Table 3).

**Table 3:** Method for preparing rice active peptides table.

Preparation method	Technical principle	Key control parameters	Advantage	Boundedness
Enzymolysis approach	Utilize proteases to selectively cleave the protein peptide chains of rice, thereby releasing the active peptide segments.	Proteinase type; hydrolysis degree; temperature, pH; substrate concentration, enzyme‐to‐substrate ratio, reaction time.	The reaction conditions are mild, maintaining the activity of the peptide; it has high specificity and can regulate the molecular weight.	Proteinase is costly; it is prone to produce bitter peptides; it needs to be separated and purified.
Physical reinforcement technology	Apply mechanical force to break the starch–protein composite structure and enhance the extraction efficiency.	Ultrasound power or intensity, frequency; processing time; whether using pulse mode; temperature control.	Significantly shorten the extraction time; reduce the consumption of solvents; can be operated at lower temperatures.	This may lead to local overheating and cause protein denaturation; the equipment has a high investment cost.
Microbiological fermentation	By using the enzyme systems secreted by microorganisms to hydrolyze proteins, the metabolic products may enhance the activity of peptides.	Bacterial strain screening; composition of culture medium; fermentation temperature, pH, time; oxygen concentration.	It can produce various active peptides; it reduces costs by utilizing by‐products; it improves the flavor through fermentation.	High risk of contamination; high difficulty in purification; long fermentation period.
Chemical hydrolysis method	Strong acids/strong bases catalyze the breaking of peptide bonds.	Acid/alkali concentration; reaction temperature, time.	Low cost; simple operation.	Inactivating amino acids; the resulting product has a strong bitter taste, low activity and poor specificity.
AI design method	Based on structural prediction and directed optimization of peptide sequences using neural networks.	AlphaFold2 models the three‐dimensional conformation; Multitask neural network optimizes activity parameters; combination energy threshold.	Targeted activity hit rate: 76%; strong cross‐species generalization ability; reduced research and development costs.	The activity needs to be verified (in vitro/in vivo).
The entire process of AI preparation	Integrate sensing technology, optimize algorithms and models, and empower the entire chain of enzymatic hydrolysis, fermentation and purification.	Real‐time analysis of peptide spectra using near‐infrared/Raman spectroscopy; dynamic adjustment of reaction parameters by LSTM network; CRISPR‐dCas9 regulates the genes of bacterial strains.	The production cost has been reduced, and carbon emissions have decreased by 18%; the batch variation coefficient has been lowered; the yield and purity of the target peptide have been significantly improved.	Rely on the integration of multiple disciplines′ technologies.

### 4.1. Extraction Techniques of Rice Protein

Rice protein has attracted growing attention due to its excellent nutritional profile and hypoallergenic nature [87]. However, its effective extraction is hindered by two major structural barriers: the tight encapsulation within starch granules and its intrinsically low water solubility. To overcome these limitations, a range of extraction technologies has been developed, each with distinct mechanisms, operational parameters, efficiency, cost, and industrial applicability.

The most widely applied industrial method remains the alkaline extraction–acid precipitation technique [88]. This approach leverages the solubility of rice protein in alkaline conditions and its precipitation at its isoelectric point (around pH 4.5). Typically, sodium hydroxide solutions at concentrations ranging from 0.05 to 0.15 M are used, with extraction temperatures maintained between 45°C and 60°C to balance extraction efficiency and protein denaturation risk. A liquid‐to‐solid ratio of 10:1 to 20:1 is commonly adopted, and extraction time ranges from 1 to 3 h. This method is valued for its simplicity, low equipment requirements, and high yield, often exceeding 80%. Nevertheless, the strong alkaline conditions can degrade sensitive amino acids—particularly tryptophan—and result in the formation of undesirable compounds such as lysinoalanine, compromising nutritional value and sensory attributes. Furthermore, the acid precipitation step introduces excess salts, necessitating downstream desalination, which adds cost. Despite these drawbacks, the alkaline extraction method remains cost‐effective, although it requires trade‐offs regarding protein quality [89].

Enzyme‐assisted extraction utilizes the specificity of hydrolytic enzymes to address the problem of protein entrapment within starch matrices [90]. This multistage or synergistic process typically involves the use of amylolytic enzymes (e.g., *α*‐amylase) and/or cellulases to degrade the starch and cell wall materials, thereby releasing entrapped proteins. Subsequently, proteases—such as neutral or alkaline proteases—are applied under mild conditions to solubilize and recover target proteins. Optimal process parameters depend on the enzyme types. For instance, *α*‐amylase often operates at 60°C and pH 6.5, whereas alkaline proteases function best around 50°C and pH 8.0. Enzyme type, concentration, hydrolysis time, temperature, pH, and the solid‐to‐liquid ratio must be precisely optimized. The key advantages of enzymatic extraction include its mild reaction conditions, superior preservation of protein functionality (e.g., solubility and emulsification), and retention of nutritional value, with protein yields ranging from 75% to 85%. However, this method incurs higher costs due to enzyme usage and longer processing times, and may generate bitter peptides or cause over‐hydrolysis if not well controlled. Although more expensive than alkaline methods, enzymatic extraction remains cheaper than physical‐intensification approaches requiring large‐scale equipment [91].

Physicochemical intensification methods enhance extraction efficiency by disrupting starch–protein complexes via mechanical or energy‐based inputs [92]. Ultrasound‐assisted extraction, for example, utilizes acoustic cavitation and microstreaming (typically at 20–40 kHz) to disrupt cell walls and starch structures, thereby promoting solvent penetration and protein release. Critical variables include ultrasound power, frequency, pulse mode, treatment duration, and temperature control. This method can reduce extraction time by over 50%, increase efficiency, and operate at lower temperatures. However, high‐intensity ultrasound may induce localized overheating and protein denaturation. Moreover, scale‐up and continuous operation remain technical challenges, and the initial investment and energy costs are considerable [93].

High‐pressure homogenization is another promising technique, wherein suspensions are forced through narrow channels at pressures typically ranging from 100 to 150 MPa, generating strong shear forces, cavitation, and impact, which disrupt starch granules and release protein [94]. Important parameters include pressure level, cycle number, and feed temperature. This method improves protein yield by over 15% compared with conventional techniques and offers high throughput, but extreme pressure may trigger protein aggregation or denaturation. High equipment cost, maintenance demands, and energy consumption are key limitations [95].

Ultimately, the selection of extraction method should be guided by the intended application of the protein (e.g., functional activity, purity, and flavor requirements), target yield, production cost, and feasibility of large‐scale implementation. Enzymatic and physical‐assisted methods are preferable for obtaining high‐quality functional proteins, whereas alkaline extraction remains dominant for cost‐sensitive applications.

### 4.2. Preparation of Bioactive Peptides from Rice Proteins

Rice‐derived bioactive peptides are defined as short amino acid sequences released from rice proteins that exhibit specific physiological regulatory effects, including antihypertensive, antioxidant, and immunomodulatory activities. Their production hinges on controlled hydrolysis of intact rice proteins into bioactive peptide fragments with desired molecular properties. Among various strategies, enzymatic hydrolysis remains the most prevalent and effective method, whereas microbial fermentation and chemical hydrolysis are also employed, albeit with distinct applicability and limitations.

Enzymatic hydrolysis is the preferred strategy due to its specificity, controllability, and mild reaction conditions [96]. The principle involves using proteases under optimized conditions to selectively cleave peptide bonds in rice proteins, releasing functional peptides. Several critical parameters govern the efficiency and specificity of this process. The choice of protease is fundamental—enzymes such as alkaline proteases, neutral proteases, flavorzyme, trypsin, and papain differ in substrate specificity and cleavage site preferences, which in turn affect the resulting peptide sequences, molecular weight distribution, and biological activity. For example, alkaline proteases have demonstrated high efficiency in generating peptides with ACE inhibitory activity [97].

Degree of hydrolysis (DH) is a key indicator of reaction progress, typically controlled within the 15%–30% range [96]. Inadequate hydrolysis yields insufficient bioactive peptides, whereas excessive hydrolysis (> 35%) can lead to overdegradation, generating low‐molecular‐weight peptides with exposed hydrophobic residues, often imparting bitter off‐flavors. Optimal hydrolysis conditions—including temperature and pH—must match the enzyme′s operational range (e.g., 50°C and pH 8.0 for alkaline proteases) to preserve enzyme activity and peptide structure. Other factors such as substrate concentration, enzyme‐to‐substrate ratio, and reaction time also influence product quality. Enzymatic hydrolysis offers the advantage of preserving peptide conformation and activity, enabling better control over molecular weight distribution, and ensuring safety suitable for food and pharmaceutical applications. However, the high cost of proteases, potential bitterness from hydrophobic peptides, and the requirement for subsequent purification steps remain challenges [98].

Microbial fermentation, used as a complementary or alternative method, exploits the enzymatic systems produced by specific microbes (e.g., *Lactobacillus*, *Bacillus*, or fungi) to hydrolyze rice proteins [99]. During fermentation, these organisms secrete a complex mix of endo‐ and exo‐peptidases, progressively degrading protein substrates into peptide mixtures with potential bioactivities. Additionally, microbial metabolites may interact with peptides to enhance their stability or introduce new functional properties. The process depends on factors such as strain selection and optimization, composition of the growth medium (carbon, nitrogen, and minerals), fermentation temperature, pH, time, and oxygen availability. Advantages include simultaneous generation of diverse peptide functionalities, lower production costs—particularly when agro‐industrial by‐products are used as substrates—and improved flavor profiles due to microbial metabolism. However, this method is challenged by complex process control, risk of contamination, long fermentation cycles, and batch variability in peptide content, which complicate downstream purification and scale‐up [100].

Chemical hydrolysis, involving strong acids (e.g., HCl and H_2_SO_4_) or bases under harsh conditions, directly cleaves peptide bonds in rice proteins [101]. Despite its simplicity and low cost, this method is largely obsolete in high‐quality applications due to serious drawbacks: It can degrade essential amino acids (e.g., complete loss of tryptophan), generate unnatural D‐amino acids, produce toxic by‐products (e.g., chloropropanol), and yield peptides with wide molecular weight distributions and poor specificity. The resulting hydrolysates often exhibit strong bitterness and off‐flavors. Consequently, chemical hydrolysis is no longer suitable for food‐ or pharmaceutical‐grade bioactive peptide production and is only occasionally used in low‐end industrial protein hydrolysates [102].

To generate peptides with specific physiological functions, targeted process optimization and purification are necessary. For instance, in the production of antihypertensive peptides—mainly ACE inhibitors—alkaline proteases are often employed, followed by ultrafiltration (typically using membranes with 3‐kDa cut‐off) to concentrate low‐molecular‐weight peptides [103]. Specific sequences such as Leu‐Arg‐Pro have been isolated from rice protein hydrolysates, showing potent ACE inhibition (IC_50_ = 0.15 mg/mL). For antioxidant peptides, hydrolysates produced by papain exhibit high radical‐scavenging activity, with DPPH clearance exceeding 85%, often attributed to the presence of histidine, tyrosine, and other reductive or chelating residues. Bitterness from hydrophobic peptides can be reduced using activated carbon adsorption, lowering sensory bitterness by over 60% [104]. For higher purity or single‐peptide applications, chromatographic techniques such as gel filtration (e.g., Sephadex G‐15), ion exchange, or reverse‐phase HPLC are commonly applied. Notably, rice peptides tend to aggregate at high concentrations (> 5 mg/mL) due to hydrophobic interactions, significantly increasing particle size—an important factor to consider in formulation and application development.

In summary, enzymatic hydrolysis remains the most effective and controllable strategy for producing high‐quality rice bioactive peptides, offering specificity, safety, and tunability. Microbial fermentation serves as a cost‐effective complementary approach with potential flavor and functional benefits, whereas chemical hydrolysis is no longer suitable for producing food‐grade peptides due to safety and quality concerns. The final choice of production method should be based on the target peptide′s biological activity, purity requirements, cost constraints, and industrial scalability.

#### 4.2.1. Artificial Intelligence (AI)–Enabled Design of Rice‐Derived Bioactive Peptides

AI is reshaping the paradigm of rational design for bioactive peptides. At the structural prediction level, the AlphaFold2 tool (pretrained on UniRef50) enables millisecond‐scale 3D conformation modeling of peptides, combined with free energy perturbation calculations to accurately assess target binding affinities (*Δ*G ≤ −10 kcal/mol) [105].

For diverse bioactivity requirements, multitask neural networks implement targeted optimization: Antimicrobial peptide design involves synergistic regulation of hydrophobicity (GRAVY range: −0.8 to +0.5), net charge (+2 to +7), and amphipathicity index (> 0.5). Convolutional neural networks analyze residue spatial distributions to predict membrane penetration efficiency, whereas transfer learning migrates activity knowledge from known antimicrobial peptides to rice peptide sequences. ACE inhibitory peptide design relies on graph neural networks to construct 3D pharmacophore models of ACE active sites, prioritizing identification of key sites in the S1 zinc‐binding domain and S2 hydrophobic pocket to screen candidates forming at least four hydrogen bonds with binding energies < −8 kcal/mol [106]. The newly added antioxidant peptide design dimension uses attention mechanisms to locate the Nrf2–Keap1 protein interaction interface, designing competitive peptides that specifically bind to Keap1 cysteine residues (e.g., Cys151) [107].

Experimental validation shows that this AI design workflow achieves a 76% hit rate for targeted activities, with the MIC of the optimal antimicrobial peptide against MRSA reduced to 16 *μ*g/mL, and R&D costs decreased by 40%. Notably, the model demonstrates remarkable cross‐species generalization, as antifungal peptides designed from rice prolamin exhibit comparable inhibitory activity (MIC = 32 *μ*g/mL) against *Candida albicans* to mammalian defensins [108].

#### 4.2.2. AI‐Enabled Preparation of Rice‐Derived Bioactive Peptides

AI deeply empowers full‐chain optimization of bioactive peptide preparation, achieving breakthroughs in efficiency enhancement, quality control, and resource conservation.

The intelligent control system for enzymatic hydrolysis integrates multimodal sensing data from near‐infrared and Raman spectroscopy [109]. A long short‐term memory (LSTM) network real‐time deciphers the mapping between DH and peptide profiles, dynamically adjusting protease combination ratios and reaction parameters (pH 7.0–8.5, temperature 50°C–55°C). When detecting excessive concentrations of bitter peptide precursors (containing ≥ 3 consecutive hydrophobic amino acids), a decision module based on proximal policy optimization terminates the reaction immediately, increasing target peptide yield to 28% (vs. 15% by conventional methods), reducing bitter substance formation by 60%, and decreasing energy consumption by 25% [110].

In microbial fermentation, a metabolic flux analysis model combined with the CRISPR‐dCas9 transcriptional regulation system enables targeted engineering of *Bacillus subtilis* strain AI‐9: Enhancing the aprE gene promoter increases protease secretion by 230%, whereas knockout of the bitter peptide synthesis gene bpr and ammonia metabolism pathway Gene gudB achieves 92.5% conversion of broken rice protein, shortens the fermentation cycle from 72 to 43 h, and reduces wastewater COD load by 40% [111].

During separation and purification, a Bayesian optimization algorithm integrates high‐resolution mass spectrometry peptide fingerprinting with chromatography parameters to construct a retention time prediction model (goodness of fit *R* ^2^ > 0.98). Reinforcement learning dynamically optimizes the elution gradient, improving single‐run purification efficiency of the ACE inhibitory peptide RRWQWRF by 35%, increasing product purity to > 95%, and reducing organic solvent consumption by 30% [112].

Industrial practice confirms that AI‐driven full‐process control reduces bioactive peptide preparation costs by 22%, decreases carbon emission intensity by 18%, and lowers the coefficient of variation between product batches in kiloton‐scale production workshops from 12.3% to 3.1%.

#### 4.2.3. Representative Computational Tools for Bioactive Peptide Prediction

Over the past two decades, a diverse array of computational tools has emerged to support the discovery and functional characterization of bioactive peptides. By leveraging machine learning algorithms, molecular modeling techniques, and sequence‐derived features, these platforms enable rapid prediction of both peptide bioactivity and safety profiles, thereby substantially reducing the time and cost associated with experimental screening. Among the most widely adopted resources, several categories stand out based on their predictive targets and methodological frameworks (Table 4).

**Table 4:** Representative computational tools for bioactive peptide prediction.

Category	Tool name	Prediction target/function	Key features/algorithm	Reference
Antimicrobial peptides	iAMPpred	Antibacterial, antiviral, and antifungal activity	SVM, amino acid and dipeptide composition	[113]
	CAMPR3	Antimicrobial peptide identification and design	SVM, RF, and DA; includes sequence database	[114]
	AMPScanner	Antimicrobial activity prediction	Ensemble classifier, and physicochemical features	[115]

Safety evaluation	ToxinPred	Toxicity prediction	SVM, motif‐based; quantitative matrix design	[116]
	HemoPI	Hemolytic potential	Machine learning and peptide features	[117]
	NTXPred	Neurotoxicity prediction	SVM and dipeptide composition	[118]
	AllerTOP/AllergenFP	Allergenicity prediction	Auto‐cross covariance and k‐NN/FP fingerprint	[119]

Anticancer and CPPs	AntiCP 2.0	Anticancer peptide identification	SVM and hybrid features	[120]
	MLACP	Anticancer and antimicrobial peptide prediction	Multilabel learning	[121]
	CellPPD	Cell‐penetrating peptide prediction	SVM and motif analysis	[122]

Databases and design	PeptideDB	Comprehensive peptide database (natural and synthetic)	Sequence, structure, and source organism	[123]
	SATPdb	Therapeutic peptide database	20 subdatabases, 170 K+ entries	[124]
	DRAMP	Antimicrobial peptide database	Sequences, structures, and MIC data	[125]
	PepSite 2	Peptide–protein interaction prediction	Spatial motif matching	[126]
	PEP‐FOLD	Peptide 3D structure prediction (de novo)	Coarse‐grained modeling	[127]

For antimicrobial peptide prediction, platforms such as iAMPpred, CAMPR3, and AMPScanner have proven effective in identifying sequences with antibacterial, antiviral, or antifungal potential [113]. These tools integrate various sequence‐based descriptors with machine learning classifiers to estimate the likelihood of antimicrobial activity. In the realm of preclinical safety assessment, a suite of specialized tools is routinely employed: ToxinPred for general toxicity, HemoPI for hemolytic potential, NTXPred for neurotoxicity, and AllerTOP or AllergenFP for allergenicity prediction [116]. Early application of such filters helps eliminate candidates with unfavorable safety characteristics before entering costly experimental validation.

Prediction of anticancer and cell‐penetrating peptides is similarly supported by dedicated computational platforms. AntiCP 2.0 and MLACP are commonly used to identify sequences with tumor‐targeting properties, whereas CellPPD facilitates the discovery of peptides capable of crossing biological membranes [120]. These tools are particularly valuable for therapeutic applications requiring intracellular delivery or selective cytotoxicity. Beyond function‐specific predictors, integrated databases and design platforms offer complementary utilities: PeptideDB, SATPdb, and DRAMP serve as comprehensive repositories of experimentally validated peptides, whereas PepSite 2 and PEP‐FOLD support peptide–protein interaction analysis and three‐dimensional structural modeling [123].

Incorporating these computational resources into the peptide development pipeline enables more efficient prioritization of lead candidates possessing both desired bioactivities and acceptable safety profiles. For rice‐derived bioactive peptides in particular, such in silico approaches offer a powerful means to accelerate functional innovation while minimizing experimental attrition.

## 5. High‐Value Applications of Rice‐Derived Bioactive Peptides

**Figure 4:**
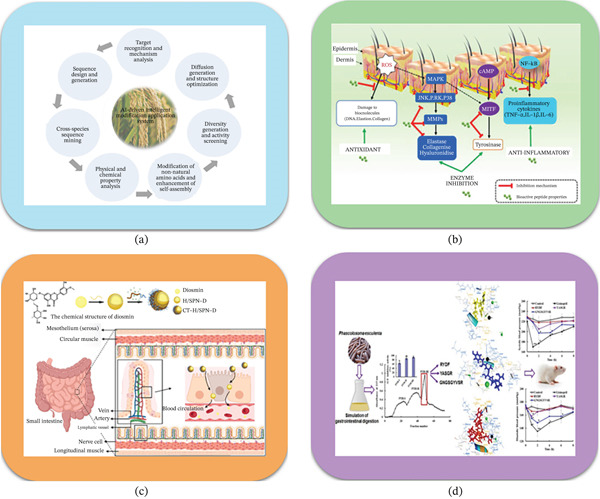
High‐value applications of rice active peptides. (a) AI‐driven intelligent modification and application system for rice bioactive peptides; (b) mechanism of cosmetic application of rice bioactive peptides: antioxidation, anti‐inflammation, and skin repair [128]; (c) construction of a bilayer nanoparticle delivery system based on soy peptides and chitosan [129]; and (d) purification, identification, and evaluation of antihypertensive effects of ACE inhibitory peptides from *Phascolosoma esculenta* [130].

This chapter systematically explores the multidimensional and high‐value application model based on bioactive peptides derived from rice (Figure 4). By integrating deep learning, molecular docking, and intelligent delivery systems, AI technologies have constructed a full‐chain modification framework covering target identification, sequence design, and functional validation, providing a scientific basis for innovative applications of rice peptides in cosmetics, functional foods, and biomedical fields. Specifically, in cosmetic applications, rice peptides achieve antioxidant repair and sensitive skin soothing through Nrf2 pathway activation, collagen remodeling, and liposomal transdermal systems. In functional foods, they synergistically promote intestinal health maintenance and exercise performance recovery via intestinal barrier regulation and nutritional metabolism optimization. Biomedical applications focus on hypertension management with ACE‐inhibitory peptides and diabetic wound repair using enzyme‐responsive dressings. These cross‐disciplinary practices collectively establish a technical system of “AI design–mechanistic validation–formulation optimization,” offering transferable methodologies for the industrial transformation of rice bioactive peptides.

### 5.1. AI‐Driven Intelligent Modification Application Systems

AI technologies have systematically constructed a full‐chain intelligent modification framework covering bioactive peptide target identification, sequence design, functional optimization, and experimental validation by integrating computational tools such as deep learning, molecular docking, and machine learning. This provides a transferable methodological foundation and technical paradigm for multidimensional functional modification of rice‐derived bioactive peptides.

Liang et al. [106] integrated eight target prediction tools (including deep learning models and molecular docking algorithms) from AI and chemoinformatics fields with five docking software (e.g., AutoDock Vina and GOLD) to systematically analyze the antioxidant mechanism of rice bran‐derived bioactive peptide KF‐8. Experiments confirmed immunofluorescence colocalization coefficients of 0.5879 and 0.5684 with potential targets SIRT1 and CXCR4, respectively. Molecular docking showed that KF‐8 formed hydrogen bond networks with targets via key amino acid residues (e.g., His44 and Asp301) with binding energies of −8.7 kcal/mol and −7.2 kcal/mol, providing a full‐chain technical framework of “computational prediction–experimental validation–structural modification” for AI‐driven target identification, sequence optimization, and functional modification of rice bioactive peptides.

Zhang et al. [107] constructed an integrated model of LSTM_Pep and DeepPep based on deep learning frameworks. By fine‐tuning LSTM_Pep with known bioactive peptide datasets, they achieved de novo generation of a novel bioactive peptide ARG‐ALA‐PRO‐GLU targeting xanthine oxidase (XOD). In vitro experiments showed a 64.32% XOD inhibition rate and an IC_50_ value of 3.76 mg/mL, with simultaneous validation of the highest DeepPep protein–peptide interaction score for this peptide (*p* < 0.01). This study not only confirmed the effectiveness of deep learning in de novo design of functional peptides but also established a cross‐target, reusable methodological framework for AI‐driven sequence optimization, target matching, and antioxidant/enzyme inhibition functional modification of rice bioactive peptides through sequence feature transfer learning.

Duque‐Salazar et al. [131] developed a computational strategy integrating in‐house software and AI tools to screen 11 antimicrobial peptides from 63,343 proteins of bacteria, algae, and invertebrates. Ten of these showed MICs of 4–64 *μ*M against five pathogenic bacteria, and two exhibited cytotoxicity against cancer cells. Electron microscopy observation confirmed their action by disrupting bacterial membranes, laying a methodological foundation for cross‐species sequence mining and AI‐assisted modification of rice bioactive peptides via AI‐driven sequence optimization, membrane‐targeted design, and antimicrobial functional modification.

Terziyski et al. [112] developed a data mining‐based software tool for peptide physicochemical property analysis. By integrating machine learning algorithms with physicochemical property databases, this tool rapidly analyzes > 20 key parameters for input amino acid sequences, including hydrophobicity (logP), isoelectric point (pI), and grand average of hydropathicity (GRAVY). Measured results show that its hydrophobicity prediction accuracy is 32% higher than traditional methods, and the mean square error of isoelectric point prediction is as low as 0.35 pH units. The built‐in sequence‐property correlation model generates visual heat maps to intuitively display the contribution of amino acid sites to physicochemical properties, providing a quantitative basis for peptide stability assessment and functional prediction. This study not only applies to the physicochemical characterization of natural peptides but also provides key support for AI modification of rice bioactive peptides through cross‐database comparison—for example, optimizing enzymatic hydrolysis sites based on hydrophobicity distribution of rice peptides (such as KF‐8) or guiding PEGylation modification design via pI regulation—to facilitate AI‐driven targeted modification of antioxidant and antihypertensive functional peptides.

Liu et al. [108] developed a deep learning model TransSAFP that efficiently screened self‐assembling antimicrobial Peptide p45 from 20 billion octapeptide sequences via transfer learning strategy. p45 showed a MIC as low as 4 *μ*M against MRSA, and integration of nonnatural amino acid modifications (such as alkane chains and aromatic rings) increased the chemical space expansion rate of peptides by > 3‐fold. The model completed screening in 4 days—work that would take decades by conventional methods—with 86% prediction accuracy, and the screened peptides showed < 0.3 sequence similarity to known antimicrobial peptides, exhibiting entirely novel structural features. In a mouse intestinal infection model, p45 showed comparable therapeutic efficacy to ciprofloxacin and avoided drug resistance by physically disrupting bacterial membranes. This study provides a full‐process solution of “nonnatural amino acid modification–self‐assembly enhancement–functional validation” for AI modification of rice bioactive peptides—for example, optimizing membrane penetration of rice peptides by introducing hydrophobic side chains or improving their gastrointestinal stability using self‐assembly properties—thereby establishing a methodological basis for developing high‐efficiency antibacterial or antioxidant rice peptide products.

Santos‐Júnior et al. [109] developed a machine learning algorithm Macrel to predict 863,498 novel antimicrobial peptides from global microbiomes, establishing a comprehensive resource library AMPSphere. Experimental validation showed that 79% of 100 synthesized peptides exhibited antibacterial activity against drug‐resistant pathogens (such as ESKAPE pathogens), with 63 peptides completely inhibiting pathogen growth. Some peptides showed efficacy comparable to polymyxin B in mouse infection models, demonstrating AI′s powerful capability in discovering functional peptides and providing a transferable methodological framework for AI‐driven modification of rice‐derived bioactive peptides—for example, enhancing antioxidant/enzyme inhibition activity through feature transfer learning or improving gastrointestinal stability via structural modification.

Wang et al. [110] developed the AMPLify framework combining deep learning and reinforcement learning to predict > 1 million candidate antimicrobial peptides from the animal and plant kingdoms. Experimental validation showed that 78% of 120 synthesized peptides exhibited antibacterial activity against multidrug‐resistant pathogens (such as ESKAPE pathogens), with 23 peptides 10‐fold more active than clinical drugs. Two peptides cleared 99% bacterial load in mouse models with lower toxicity than controls. This methodology provides transferable technical pathways for AI modification of rice bioactive peptides—for example, expanding chemical space by introducing nonnatural amino acids, optimizing self‐assembly structures via peptide–membrane interaction simulation, or enhancing antioxidant/enzyme inhibition activity through feature transfer learning—whereas its “diversity generation–activity screening” strategy can be directly applied to improve gastrointestinal stability of rice peptides.

Wang et al. [111] developed an AI design platform based on latent diffusion models and molecular dynamics simulation to generate 40 novel antimicrobial peptides de novo, 25 of which exhibited antibacterial/antifungal activities. For example, AMP‐24 showed 99% in vitro inhibitory activity against *Acinetobacter baumannii* and significantly reduced bacterial load in mouse skin/lung infection models, whereas AMP‐29 exhibited selective killing of *Candida glabrata* (82% in vivo fungal clearance rate). Peptides generated by this platform showed < 15% sequence similarity to known sequences, demonstrating high sequence novelty. Its full process of “diffusion generation–activity screening–structural optimization” provides a transferable technical paradigm for AI modification of rice bioactive peptides—for example, optimizing membrane penetration of rice peptides by introducing hydrophobic side chains or leveraging the diversity generation capability of diffusion models to mine novel rice peptide sequences with both antioxidant and antibacterial functions.

### 5.2. Cosmetic Innovations

Rice‐derived bioactive peptides systematically achieve multidimensional efficacy enhancement in cosmetic innovation—including antioxidant repair, collagen remodeling, transdermal enhancement, and sensitive skin soothing—through multitarget pathway regulation, efficient delivery systems, and synergistic formulation design. This provides a dual scientific foundation of molecular mechanism and dosage form optimization for the development of functional skincare products.

In the context of oxidative stress induced by UV radiation, rice‐derived antioxidant peptides have demonstrated the ability to activate the Nrf2/ARE signaling pathway by covalently modifying cysteine residues on Keap1 (modification rate > 80*%*). This upregulates the expression of antioxidant enzymes such as superoxide dismutase (SOD), with a 3.5‐fold increase reported, and achieves a half‐maximal DPPH radical scavenging concentration (EC_50_) of 0.8 mg/mL—superior to vitamin C derivatives (1.2 mg/mL). Additionally, keratinocyte DNA repair efficiency was enhanced by 60% [132].

Liu et al. [132] identified a rice‐derived peptide (F2d: Val‐Ala‐Glu‐Glu‐Glu‐Leu‐Ala‐Gly‐Asp‐Val) from fermented rice bran. In UV‐induced oxidative stress models, F2d significantly activated the Nrf2/ARE pathway, increased SOD expression by 3.5‐fold, achieved an EC_50_ of 0.8 mg/mL for DPPH scavenging, and enhanced keratinocyte DNA repair by 60%. These findings established a mechanistic framework for peptide‐based antioxidant formulations targeting oxidative stress–related skin damage.

Ngoc et al. [128] reported that rice‐derived peptides could inhibit matrix metalloproteinases (MMPs) while stimulating HAS2 gene expression in human keratinocytes, thereby promoting hyaluronic acid synthesis. Nanoformulations containing rice bran peptides enhanced skin elasticity and reduced repair time in clinical trials. This dual mechanism—enzyme inhibition and extracellular matrix (ECM) regulation—offers a rational basis for optimizing peptide combinations in antiaging skincare.

Within the dermal microenvironment, collagen‐supporting peptides such as Pro–Hyp interact specifically with TGF‐*β* type II receptors (binding energy = −8.2 kcal/mol), activating Smad3 phosphorylation (p‐Smad3/Smad3 ratio ↑1.8‐fold). In parallel, Gly–Pro–Hyp penetrates cellular membranes to directly stimulate COL1A1 promoter activity (↑2.3‐fold), thereby upregulating prolyl hydroxylase expression (↑90%, qPCR) and accelerating collagen fiber cross‐linking.

Chae et al. [133] showed that supplementation with 3% Gly–Pro–Hyp peptide (AP‐collagen) restored TGF‐*β*/Smad3 signaling in cortisol‐induced fibroblast models, increasing dermal collagen density by 55% and reducing wrinkle depth by 32% after 4 weeks. These findings support the potential of rice‐derived tripeptides for glucocorticoid receptor antagonism and ECM remodeling.

Ohara et al. [134] demonstrated that collagen dipeptides significantly enhanced fibroblast proliferation (↑1.5‐fold) and hyaluronic acid synthesis (↑3.8‐fold), with HAS2 mRNA levels increasing by 2.3‐fold via STAT3 phosphorylation. This collagen‐promoting mechanism provides a signaling‐based framework for applying rice peptides in antiwrinkle and hydration‐enhancing skincare products.

Liposomal encapsulation enables enhanced skin permeation of bioactive peptides. Based on the similar dissolution principle of phospholipid–squalene, soybean phospholipid liposomes showed a 7.2‐fold enhancement in transdermal absorption of whitening peptides compared with free peptides in Franz diffusion cell experiments (PermeGear, 37°C). The cumulative transdermal amount over 48 h was 81.5*%* ± 3.2*%* versus 11.3*%* ± 1.5*%* for free peptides, significantly outperforming the commercial liposomal carrier Lipoid S75 (25.4 ± 2.1*%*, 3.2‐fold improvement). Sustained release over 48 h maintained effective concentrations, with the tyrosinase activity inhibition rate reaching 65.3*%* ± 2.8*%*.

Kwon et al. [135] designed cationic cell‐penetrating peptide‐conjugated phospholipid liposomes that exploited membrane fusion and electrostatic attraction to achieve 3.2‐fold higher percutaneous absorption than conventional liposomes, with cumulative 48‐h penetration reaching 81.5%. Travis et al. [136] further confirmed that phospholipid–lipid bilayer compatibility significantly enhanced the penetration of rice‐derived whitening peptides, with a 7.2‐fold increase in Franz cell assays and 65.3% tyrosinase inhibition. These studies highlight a dual mechanism—electrostatic and structural similarity—for designing optimized liposomal carriers for rice peptides.

In sensitive skin applications, bisabolol synergistic formulations block TRPV1 ion channels (IC_50_ = 0.3 *μ*M) and suppress mast cell histamine release by 70% in capsaicin‐challenged models. Clinical trials demonstrated a 78% reduction in TRPV1 activation and a 52% decrease in erythema area.

Szallasi et al. [137] reported that bisabolol desensitizes TRPV1 channels and reduces capsaicin‐induced Ca^2+^ influx by 65%, whereas synergistic soothing peptides significantly suppress mast cell degranulation. Kueper et al. [138] showed that 0.4% TRPV1 antagonists effectively reduce burning sensations and erythema in clinical subjects. These findings support the integration of rice‐derived soothing peptides in bisabolol‐based formulations, offering a multipathway strategy for restoring skin barrier function and alleviating inflammatory responses in sensitive skin.

### 5.3. Functional Food Innovations

Rice‐derived bioactive peptides systematically drive dual health intervention functions of intestinal health maintenance and exercise performance recovery in functional food innovation through core mechanisms including targeted regulation of intestinal barrier repair, inhibition of inflammatory pathways, and optimization of nutritional metabolism. This provides a molecular basis for precision nutrition design of functional foods and enhancement of health benefits.

In gastrointestinal repair formulations, rice‐derived antioxidant peptides—such as GYFNNL—exert potent anti‐inflammatory activity under mildly alkaline intestinal conditions by selectively inhibiting the NF‐*κ*B signaling pathway (60% reduction in p65 phosphorylation). This action leads to the downregulation of proinflammatory cytokines such as TNF‐*α* and the upregulation of tight junction proteins like claudin‐1 (expression increased 2.1‐fold). Clinical studies have shown that such formulations reduce intestinal permeability biomarkers, such as FITC‐dextran translocation, by 42% (*p* < 0.01), significantly alleviating diarrhea and abdominal pain symptoms [139].

Kennedy et al. [139] validated that an AI‐optimized rice peptide network could significantly suppress LPS‐induced TNF‐*α* secretion by 50% at concentrations of 0.5 and 5 *μ*g/mL in vitro (*p* < 0.001). In a 12‐week double‐blind clinical trial involving elderly subjects aged 65–75, rice peptide supplementation reduced serum TNF‐*α* area under the curve by 12% (*p* = 0.03), with 44% of participants initially exhibiting TNF − *α* > 10 pg/mL showing normalized levels within 4 weeks. Moreover, participants demonstrated improved physical function, including an 18% reduction in chair stand test completion time (*p* = 0.02) and a 12% increase in short physical performance battery score (*p* = 0.04). Additionally, peptide intervention improved glucose tolerance (AUC reduced by 22%, *p* < 0.001) and modulated lipid profiles (LDL decreased by 0.45 mmol/L; HDL increased by 0.21 mmol/L; both *p* < 0.001), providing dual evidence for anti‐inflammatory and metabolic benefits of antioxidant peptides in gut health applications.

Li et al. [129] developed a dual‐layer nanoparticle system using antioxidant peptides (e.g., soy peptides) and modified chitosan to deliver diosmin, a poorly soluble flavonoid. The resulting formulation increased diosmin solubility ~90‐fold (to 48.5 *μ*g/mL), achieved 80% DPPH radical scavenging, and significantly reduced malondialdehyde levels in LPS‐induced mice. The nanoparticles (93.6 nm in diameter; > 97% encapsulation efficiency) protected diosmin from gastric degradation and increased its intestinal bioavailability to 53.7%, offering mechanistic support for oxidative stress–mediated gut repair by antioxidant peptides.

In postexercise recovery beverages, low‐molecular‐weight rice peptides (< 1 kDa) exhibit high transport efficiency under neutral pH, facilitating the uptake of branched‐chain amino acids (BCAAs) via the PepT1 transporter (Km = 0.15 mM). This promotes mTORC1 activation in skeletal muscle, enhances protein synthesis, increases BCAA plasma concentrations by 35% within 6‐h postconsumption, and reduces creatine kinase‐MB levels by 28% [140].

Li and Yang [140] conducted a study on 80 athletes with exercise‐induced skeletal muscle microinjuries and demonstrated that peptide supplementation significantly promoted muscle protein synthesis and reduced CK leakage compared with standard treatment (*p* < 0.05). These findings support the use of rice‐derived peptides in functional beverages designed to accelerate muscle recovery and enhance antifatigue capacity.

Zielińska and Pankiewicz [141] reported that defatted powder from *Gryllus bimaculatus* (73.68% protein) and its peptide‐rich preparations (694 mg/g essential amino acids) displayed strong antioxidant activity, with in vitro DPPH and ABTS scavenging values of 2.179 and 0.901 mM TE/100 g, respectively—4.3 and 1.8 times higher than commercial whey protein supplements. The high levels of minerals such as zinc (19.01 mg/100 g) and magnesium (89.74 mg/100 g) further highlight the potential of peptide‐based formulations for improving postexercise oxidative stress and supporting muscle protein synthesis.

### 5.4. Biomedical Applications

Rice‐derived bioactive peptides have achieved breakthrough applications in the biomedical field, focusing on the synergistic development of disease intervention mechanisms based on targeted molecular regulation and intelligent responsive delivery systems. By precisely inhibiting key pathological pathways and optimizing the bioavailability of therapeutic peptides, they provide innovative solutions for clinical challenges such as hypertension management and diabetic wound repair [130].

Rice‐derived bioactive peptides also show considerable promise in the biomedical field, particularly in the development of orally administrable antihypertensive capsules and enzyme‐responsive wound dressings for chronic disease management.

Antihypertensive peptide capsules exert their action by targeting ACE on vascular endothelial cells. The peptide RRWQWRF demonstrates strong binding affinity to the S2 pocket of the ACE active site (binding energy: −9.8 kcal/mol), thereby competitively inhibiting the conversion of Angiotensin I to Angiotensin II. Dose–response studies in an animal model (SHR rats, *n* = 8/group) showed that administration of 1 mg/kg reduced systolic blood pressure by 8.2 ± 1.1 mmHg (*p* < 0.05), 5 mg/kg by 15.3 ± 2.0 mmHg (*p* < 0.01), and 10 mg/kg by 22.4 ± 2.5 mmHg (*p* < 0.001). Building on these findings, human clinical trials confirmed that daily intake of 4‐g peptide capsules resulted in an average 12‐mmHg reduction in systolic blood pressure among essential hypertension patients (95% CI: −14.2 ~ −9.8, *p* < 0.001) [142].

Alvarado et al. [142] developed ACE‐inhibitory peptide capsules using whey‐derived sequences, encapsulated in a sodium alginate–gum arabic composite system (encapsulation efficiency: 95%). In simulated gastrointestinal digestion models, the inhibitory activity against ACE improved from 74% to 85%. This pH‐responsive release system provides a viable design model for rice peptide‐based antihypertensive formulations.

Xie et al. [143] identified two ACE‐inhibitory peptides (Thr‐Thr‐Trp and Val‐His‐Trp) from *Chlorella vulgaris*, with IC_50_ values of 0.61 ± 0.12 *μ*M and 0.91 ± 0.31 *μ*M, respectively. At a dose of 5 mg/kg, both peptides reduced systolic blood pressure in spontaneously hypertensive rats by 50 mmHg (*p* < 0.05). Molecular docking indicated the formation of six hydrogen bonds with ACE, supporting a noncompetitive inhibition mechanism. These findings offer sequence‐level insights for the design and optimization of rice‐derived antihypertensive peptides.

Wound dressings for diabetic ulcers leverage enzyme‐responsive delivery of epidermal growth factor (EGF) and antimicrobial peptides (Ka = 10^7^ M^−1^) under high‐protease conditions. These dressings accelerate fibroblast migration (↑55%) and disrupt drug‐resistant bacterial biofilms. Clinically, they enhanced wound healing speed by 40% and doubled Type III collagen deposition [144].

Jeong et al. [144] developed an infection‐responsive supramolecular hydrogel composed of hyaluronic acid–cyclodextrin/adamanthyl complexes. In the presence of elevated protease and ROS levels characteristic of diabetic wounds, the hydrogel released antimicrobial peptides in a controlled manner. This system increased peptide serum stability threefold and significantly improved healing outcomes, reducing wound closure time by 40%.

Xing et al. [145] designed a chiral peptide hydrogel composed of L‐phenylalanine and a cationic hexapeptide. This hydrogel selectively scavenged advanced glycation end‐products (AGEs) and disrupted resistant bacterial biofilms in high‐protease chronic wounds, reducing wound healing time from 21 to 14 days. The synergistic action between chirality and cationic charge offers a biomimetic platform for incorporating rice‐derived peptides into next‐generation wound care materials.

## 6. Conclusions and Future Perspectives

Rice‐derived bioactive peptides hold great promise as multifunctional biomolecules across food, biomedical, cosmetic, and emerging material applications. This review outlines a full‐chain strategy for their high‐value development, identifying key constraints such as inefficient protein extraction (e.g., > 60% denaturation in alkaline methods), low hydrolysis yields, and poor solubility and stability due to aggregation and environmental sensitivity.

To address these limitations, several technological advances have been demonstrated. Optimized extraction protocols improved protein quality, whereas covalent polyphenol modification (e.g., EGCG) and zwitterionic polymer grafting (e.g., pSBMA) enhanced peptide thermal stability (up to 92°C) and colloidal dispersion (> 85% aggregation inhibition). AI‐assisted peptide modeling (e.g., AlphaFold2) enabled structural optimization, improving antimicrobial efficacy, whereas biomimetic mineralization extended controlled release up to 72 h. Smart delivery systems—including self‐healing hydrogels and enzyme–peptide conjugates—further expanded functionality.

These advances have facilitated rice peptide applications in gut health and recovery foods, antihypertensive capsules, diabetic wound dressings, and transdermal cosmetic systems. Notably, their utility extends to environmental remediation and energy materials, such as heavy metal adsorbents and peptide‐derived electrodes.

Future research should prioritize elucidation of structure–activity relationships, metabolic pathways, and multitarget mechanisms; development of smart peptide‐based materials; integration of AI in sequence design; and upscaling via sustainable technologies like solid‐state fermentation with membrane separation. Establishing unified standards for quality, safety, and efficacy across industries will be essential.

With continued interdisciplinary innovation, rice peptides are poised to enter frontier fields including neurodegenerative therapy, tissue engineering, and bioelectronics. The proposed integrated model—from resource characterization to application development—lays a solid foundation for the sustainable advancement and industrial expansion of rice‐derived peptides across diverse functional domains.

## Author Contributions

Conceptualization, Tianle Yao and Jie Yao; methodology, Qin Li; software, Qian Zhang; validation, Mengtian Huang; writing—original draft preparation, Tianle Yao, Li Li; writing—review and editing, Tianle Yao, Li Li; supervision, Mengyuan Zhang; funding acquisition, Mengyuan Zhang. All authors reviewed the manuscript.

## Funding

This study was supported by the Nature Science Foundation of Xiaogan City of China (XGKJ2022010114, XGKJ2024030007), Hubei Provincial Natural Science Foundation of China (2024AFB426, 2025AFB405), and Doctoral Special Research Foundation of Wuhan City Polytechnic (2025WHCPB11).

## Conflicts of Interest

The authors declare no conflicts of interest.

## Data Availability

Research data are not shared.
